# Peripheral inflammation relationships with cognitive deficits and genetic factors in psychosis

**DOI:** 10.1192/j.eurpsy.2023.153

**Published:** 2023-07-19

**Authors:** L. Zhang

**Affiliations:** Experimental and Clinical Pharmacology, University of Minnesota, Minneapolis, United States

## Abstract

**Abstract:**

Elevated peripheral inflammation is common in psychosis. Impairments in general cognition were linked to elevated C-reactive protein (CRP) and other inflammatory markers in patients with psychotic disorders. Whether there is a subgroup of persons with elevated peripheral inflammation demonstrating deficits in specific cognitive domains remains unclear. While molecular underpinnings of altered inflammation in psychosis are hypothesized, genetic contributions to relationships of psychosis, inflammation, and cognition have not been clarified. Thirteen peripheral inflammatory markers and 17 neurobehavioral tasks were quantified in a subset of participants (129 psychosis, 55 healthy controls-HCs) from the Bipolar-Schizophrenia Network on Intermediate Phenotypes (B-SNIP) consortium. Principal component analysis resulted in 5 inflammation factors across inflammatory markers. Three latent cognitive domains (Visual Sensorimotor, General Cognitive Ability, and Inhibitory Control) were characterized based on the neurobehavioral battery. Hierarchical clustering identified a psychosis subgroup with elevated inflammation and worse cognitive performance. Genetic predispositions to schizophrenia and cognition were explored in relation to inflammation. Among persons with psychosis, higher inflammation indices were associated with impairments of Inhibitory Control and Visual Sensorimotor function. Greater deficits in Inhibitory Control were observed in a high inflammation patient subgroup. Consistent with previous studies, global genetic correlations of schizophrenia, CRP, and cognition were observed. Significant bivariate local genetic correlations of CRP with schizophrenia or cognition across 22 loci with several genes in 1 locus on chromosome 3 suggested pleiotropic mechanisms for inflammatory relationships with cognition and psychosis. Specific neurobehavioral domains may be more sensitive to inflammation dysregulation in psychosis as compared to general cognitive function, particularly performance on tasks requiring ongoing behavioral monitoring and control. These, along with evidence of genetic correlations of CRP, psychosis, and cognition, provide further supporting evidence that inflammation dysregulation is an important underlying mechanistic contributor to the disruption of cognition in psychosis. Targeting this dysregulation may be an avenue for novel therapeutics to improve cognitive outcomes in these patients.

**Disclosure of Interest:**

None Declared

